# Apelin inhibits the proliferation and migration of rat PASMCs *via* the activation of PI3K/Akt/mTOR signal and the inhibition of autophagy under hypoxia

**DOI:** 10.1111/jcmm.12208

**Published:** 2014-01-22

**Authors:** Hongyu Zhang, Yongsheng Gong, Zhouguang Wang, Liping Jiang, Ran Chen, Xiaofang Fan, Huanmian Zhu, Liping Han, Xiaokun Li, Jian Xiao, Xiaoxia Kong

**Affiliations:** a School of Pharmacy, Zhejiang Key Laboratory of Biotechnology and Pharmaceutical Engineering, Wenzhou Medical UniversityWenzhou, Zhejiang, China; b School of Basic Medical Sciences, Institute of Hypoxia Research, Wenzhou Medical UniversityWenzhou, Zhejiang, China; c School of Nursing, Wenzhou Medical UniversityWenzhou, Zhejiang, China

**Keywords:** apelin, hypoxia, autophagy, PI3K/Akt/mTOR, smooth muscle cells

## Abstract

Apelin is highly expressed in the lungs, especially in the pulmonary vasculature, but the functional role of apelin under pathological conditions is still undefined. Hypoxic pulmonary hypertension is the most common cause of acute right heart failure, which may involve the remodeling of artery and regulation of autophagy. In this study, we determined whether treatment with apelin regulated the proliferation and migration of rat pulmonary arterial smooth muscle cells (SMCs) under hypoxia, and investigated the underlying mechanism and the relationship with autophagy. Our data showed that hypoxia activated autophagy significantly at 24 hrs. The addition of exogenous apelin decreased the level of autophagy and further inhibited pulmonary arterial SMC (PASMC) proliferation *via* activating downstream phosphatidylinositol-3-kinase (PI3K)/protein kinase B (Akt)/the mammalian target of Rapamycin (mTOR) signal pathways. The inhibition of the apelin receptor (APJ) system by siRNA abolished the inhibitory effect of apelin in PASMCs under hypoxia. This study provides the evidence that exogenous apelin treatment contributes to inhibit the proliferation and migration of PASMCs by regulating the level of autophagy.

## Introduction

Hypoxic pulmonary hypertension (HPH) is a severe disease characterized by pulmonary vasoconstriction, pulmonary arterial remodeling and abnormal angiogenesis. Hypoxic pulmonary hypertension eventually leads to right ventricular pressure overload, which is the most common cause of acute right heart failure [1–3]. It has been reviewed extensively that proliferation of smooth muscle cells (SMCs) is the key event in the pathogenesis of HPH [4]. Indeed, SMC proliferation in small, peripheral and normally non-muscular pulmonary arterioles is a hallmark of HPH [5,6]. The remodeling of the pulmonary artery also involves proliferation and migration of SMCs induced by hypoxic insult [7,8], but the abnormal proliferation mechanisms of vascular SMCs (VSMCs) are still a source of controversy.

Autophagy is a dynamic process in the turnover of organelles and proteins through a lysosome-associated degradation process, and serves a critical function in cellular homoeostasis by regulating cell survival and cell death pathways [9]. To date, autophagy has been implicated in development and several other human diseases [10], including cancer [11,12], neurodegenerative diseases [13], inflammatory diseases [14] and cardiovascular diseases [15]. However, very little is currently known about the physiological function of autophagy in the clinical progression of human pulmonary diseases. In general, autophagy could signify at least two possible functions. First, autophagy represents an early adaptive mechanism of the tissue to the clearing of damaged organelles or proteins for regenerating nutrients and energy and restoring tissue homoeostasis. Moreover, excess autophagy is considered to contribute to cell death [9,^11^,16]. It has been demonstrated that autophagy is activated as a protective response under hypoxic conditions in several cancer cells [17–19]. The molecular mechanism of autophagy is complex and involves several distinct signal pathways. Most of all, the phosphatidylinositol-3-kinase (PI3K)/protein kinase B (Akt)/the mammalian target of Rapamycin (mTOR) signalling pathways negatively regulate autophagy under certain conditions [20,21]. However, the role of autophagy has still not been elucidated completely in HPH.

The peptide apelin is a recently described ligand for the G-protein–coupled receptor APJ (APLNR). Both apelin and apelin receptor (APJ) are highly expressed in the lungs, especially in the endothelium of the pulmonary vasculature [22,23]. As a potential biomarker for HPH, the peptide regulates the proliferation of VSMCs, vasodilator function and positive inotropic effects [24]. The expression of apelin and APLNR is regulated by hypoxia-induced factor 1α and has been shown to be involved in normal vascular development and the regulation of apoptosis [25]. Furthermore, the activation of PI3K/Akt/mTOR signalling pathways is also involved in the effects of apelin [26]. Although high levels of expression of the APJ receptor and apelin in the lungs are observed [27], the functional role of these proteins during normal lung development and under pathological conditions such as HPH is still undefined.

In this study, we investigated the effect of exogenous apelin in a HPH cell model *in vitro*. Our data indicate that hypoxia stimulated the proliferation and migration of primary cultured pulmonary arterial SMCs (PASMCs) *via* the activation of autophagy. The addition of exogenous apelin decreased the level of autophagy and further inhibited PASMCs proliferation. Thus, the mechanism of apelin may involve the activation of downstream PI3K/Akt/mTOR signal pathways. The inhibition of the APJ system by siRNA enhanced the proliferation and autophagy of PASMCs under hypoxia. To the best of our knowledge, this study provides the novel evidence that the application of apelin may provide potential therapeutic strategy, targeting of the inhibition of autophagy and artery remodeling in HPH.

## Materials and methods

### Cell culture

Primary PASMCs were derived from micro-dissected segments of pulmonary arteries as described previously [28]. Lung tissues were obtained from a 3-month-old healthy Wistar rat. Cells were cultured in DMEM (Invitrogen, Carlsbad, CA, USA) supplemented with 10% fetal bovine serum (FBS), 100 IU/ml penicillin and 100 μg/ml streptomycin at 37°C in a 5% CO_2_/95% air environment. Cells (passages 3–10) were phenotyped using immunohistochemical and receptor-binding techniques and, like the SMCs in the medial layer of pulmonary arteries, they expressed smooth muscle actin [29]. Cells were incubated for at least 24 hrs in serum-free DMEM prior to treatment with apelin at stated concentrations.

A hypoxia chamber was placed in a regular CO_2_ incubator maintained at 37°C. The concentration of oxygen in the chamber was monitored with an oxygen analyser, showing stable oxygen concentration as indicated on the cylinders. Pulmonary arterial SMCs were exposed to 1% oxygen for different time-points and then harvested for cell proliferation assay and cell cycle analysis. Pulmonary arterial SMCs under normoxia were also established as controls.

### RNA interference construction

Plasmids were purified with a HiSpeed Plasmid Maxi Kit (Qiagen Inc., Hilden, Germany). The used mouse apelin siRNA (National Center for Biotechnology Information, accession numbers NM_031349) corresponded to the following cDNA sequence: 5′-AAGAGACGCTCAGCTGACA-3′. The pSUPER neo RNAi plasmid was purchased from OligoEngine (Seattle, WA, USA). siRNAs were transfected into PASMCs using Lipofectamine 2000 Transfection Reagent (Invitrogen) according to the manufacturer's recommendations as described previously [30]. The knockdown efficiency for apelin was determined by western blot analysis. After 24 hrs, the transfected cells were ready for experimental use.

### Cell proliferation and cell cycle assays

Cell proliferation was also assessed by incorporation of the thymidine analogue 5-bromo-2′-deoxyuridine (BrdU) into the DNA of replicating cells using a commercially available colorimetric immunoassay according to the recommended protocol (Invitrogen). The cell proliferation capacity was recorded as a percentage of BrdU-positive nuclei over the total nucleated cells.

For cell cycle analysis, PASMCs were incubated, treated and then harvested. Pellets of the cells were obtained by centrifugation. Following removal of the media, the pellets were resuspended with 10 μl of PBS, and 1 ml of 70% ethanol was added followed by centrifuging and washing with cold PBS. The cells were then resuspended in 20 μg/ml of propidium iodide/PBS with 1 mg/ml of RNase. After incubating for 15 min. at room temperature, the samples were then analysed using a FACScan flow cytometer (Becton Dickinson, Franklin Lakes, NJ, USA).

### Cell migration assay

A wound healing migration assay with PASMCs was performed following previously published methods [31]. Briefly, the cells were seeded at 4 × 10^6^ cells/well on 12-well cell culture plates. On the second day, three straight scratches for each well were made with a 200-μl pipette tip. The wells were then rinsed with PBS, which was replaced with regular media, and the cells were incubated in less than 1% oxygen for 24 hrs. Cell migration was captured with a light microscope. A migration assay was also performed with a modified Boyden chamber with a transwell pore size of 8 μm. The cells were trypsinized, counted and then seeded into the upper insert at densities of 1 × 10^5^ cells/24-well in serum-free DMEM. DMEM containing 10% FBS was added to the lower chamber. The cells were incubated for 24 hrs under normoxia or hypoxia. The counting of migrated cells was performed after fixation and trypan blue staining of cellular nuclei.

### Cell apoptosis analysis

To determine the effect of exogenous apelin on hypoxic PASMCs, cells were seeded on 6-well plates (2 × 10^5^ cells/well) and exposure to hypoxia as described above or treated with apelin. Then, cells were washed and harvested; apoptotic rates were measured using a PI/Annexin V-FITC kit (Invitrogen) and analysed by FACScan flow cytometer (Becton Dickinson).

### Immunofluorescence staining analysis

The level of autophagy is characterized by the development of autophagic vacuoles. Monodansylcadaverine (MDC) has been proposed as a tracer for autophagic vacuoles [32]. Pulmonary arterial SMCs were cultured on coverslips overnight, treated with different stimuli doses for 24 hrs as described above and rinsed with PBS. They were then stained with 50 μM MDC at 37°C for 1 hr. After incubation, the cells were fixed for 15 min. with ice-cold 4% paraformaldehyde at 4°C. In addition, for immunocytochemical analysis, immunocytochemical analysis of cells cultured on coverslips was performed. Briefly, the coverslips were fixed with 4% paraformaldehyde in PBS for 20 min., permeabilized with 0.2% Triton X-100 in 0.1 M PBS for 5 min., blocked in 10% goat serum for 30 min. and incubated overnight at 4°C with polyclonal antibodies to LC3 (Santa Cruz Biotechnology, Santa Cruz, CA, USA). After washing three times with 0.1 M PBS (pH 7.4), the cells were incubated with fluorescence-conjugated secondary antibody (Sigma-Aldrich, St. Louis, MO, USA) for 90 min. at room temperature and examined using a Nikon ECLIPSE Ti fluorescence microscope (Nikon, Tokyo, Japan).

### Immunoblotting

Cells were harvested after different treatment as described above, washed with cold PBS and incubated in ice-cold RIPA buffer. The cell lysates were sonicated for 30 sec. on ice and then incubated at 4°C for 60 min. The lysates were centrifuged for 30 min. at 12,000 × g, and the protein concentration was assessed with the BCA protein assay (Thermo Scientific, Rockford, IL, USA). For Western blot analysis, lysate proteins (30 μg) were resolved using 8%, 10% and 12% SDS-PAGE and transferred to nitrocellulose by electroblotting. Non-specific binding sites were blocked with 5% non-fat dry milk in buffer (10 mM Tris-HCl [pH 7.6], 100 mM NaCl and 0.1% Tween 20) for 1 hr at room temperature and then incubated with the desired primary antibody (all from Santa Cruz Biotechnology) overnight at 4°C, followed by incubation with horseradish peroxidase–conjugated secondary antibody at a 1:2000 dilution for 1 hr at room temperature. The immunoreactive bands were visualized using the diaminobenzidine (Sigma-Aldrich) coloration method.

### Statistical analysis

The results are expressed as the mean ± SEM. Statistical significance was determined with Student's *t*-test when there were two experimental groups. For more than two groups, statistical evaluation of the data was performed with the one-way anova test, followed by Dunnett's multiple-comparisons test. A value of *P* < 0.05 was considered the minimum level of statistical significance.

## Results

### Hypoxia increases proliferation and migration of cultured pulmonary artery SMCs

**Figure 1:**
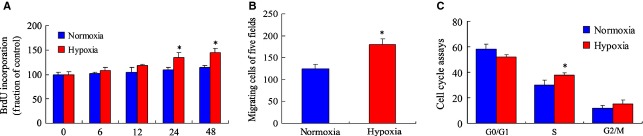
Hypoxia increases the proliferation and cell cycle progression of pulmonary arterial smooth muscle cells (PASMCs). (A) PASMCs were seeded at 1 × 10^4^ cells/well (0.1 ml) in 96-well flat-bottomed plates and incubated overnight at 37°C. After exposure to hypoxia (1% oxygen) and normoxia chamber, respectively, for 6, 12, 24 and 48 hrs, cell proliferation was measured by 5-bromo-2′-deoxyuridine (BrdU) incorporation. The values are mean ± SD,*n* = 5. (B) Cell migration of PASMCs under hypoxia condition at 24 hrs by transwell assays. Columns represent the mean of three individual experiments performed in triplicate. *P < 0.05 *versus* normoxia group. (C) Cell cycle analysis of PASMCs in hypoxia condition at 24 hrs by flow cytometry. The results were expressed as relative cell growth in percentage, which was compared with a 21% oxygen control group. The concentration of 21% oxygen was set as control. *n* = 5 for each group. *P < 0.05 *versus* normoxia group.

To mimic the hypoxia-induced proliferation of pulmonary arterial SMCs *in vivo*, primary cultured PASMCs were incubated for different times (6, 12, 24 and 48 hrs) at 1% oxygen concentration in the hypoxia chamber with the 21% oxygen of the room air being used for controls. The cells were harvested for proliferation assays and cell cycle analysis. According to the BrdU incorporation assay, cell proliferation increased obviously from 24 hrs under hypoxia as compared with the normoxia group (*P* < 0.05, Fig. 1A). Moreover, the migration ability of PASMCs was examined using a cell migration assay. The number of migrated cells increased significantly at 24 hrs in response to hypoxia compared with the normoxia group (*P* < 0.05, Fig. 1B). Subsequently, the cell cycle was analysed with flow cytometry. Our data indicate that enhanced transitions from the G1 into the S phase were measured under hypoxic conditions (*P* < 0.05, Fig. 1C). These results indicate that the proliferation, migration and the cell cycle progression of PASMCs were stimulated by hypoxia treatment.

### The enhancement of PASMCs proliferation is related to the activation of autophagy in response to hypoxia

**Figure 2:**
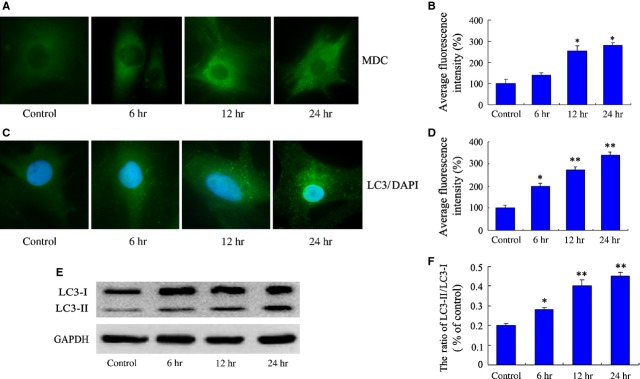
Activation of autophagy in pulmonary arterial smooth muscle cells (PASMCs) under hypoxia. (A) Monodansylcadaverine (MDC) fluorescence staining of autophagic vacuoles in PASMCs treated with hypoxia condition. (B) The corresponding linear diagram of MDC staining results. (C) Representative immunofluorescence images of PASMCs stained with DAPI (blue) for nucleus and antibodies against LC3 (green) for autophagosomes; punctuated LC3 dots were considered as positive results. Images are at 1000×. (D) The corresponding linear diagram of LC3 staining. (E) The levels of LC3-II and LC3-I were measured in the PASMCs under hypoxia by western blot analysis. Similar results were observed in three independent experiments. (F) The ratio of LC3-II to LC3-I was normalized to GAPDH. The data were presented as a mean ± SD from three independent experiments. *P < 0.05 *versus* control group, **P < 0.01 *versus* control group.

To demonstrate whether autophagy was involved in the process that hypoxia increases proliferation of PASMCs, cells were cultured in hypoxia chamber for different time-points (6, 12 and 24 hrs), and autophagic vacuoles were detected by MDC staining. As shown in Figure 2A and B, the accumulation of MDC-positive dots was obviously increased under hypoxia from 6 hrs as compared with the normoxia control group. In LC3 immunofluorescence staining analysis, the formation of LC3 puncta, representing autophagosomes, was extensively induced in cells exposed to hypoxia at 6 hrs (Fig. 2C and D). The level of autophagy was also determined by western blot analysis. The expression of autophagic protein, microtubule-associated protein-1 light chain-3-II (LC3-II), increased significantly from 6 hrs (Fig. 2E and F). These results indicate that autophagy was activated in the early stage of hypoxic stimulation with a time-dependent increase.

**Figure 3:**
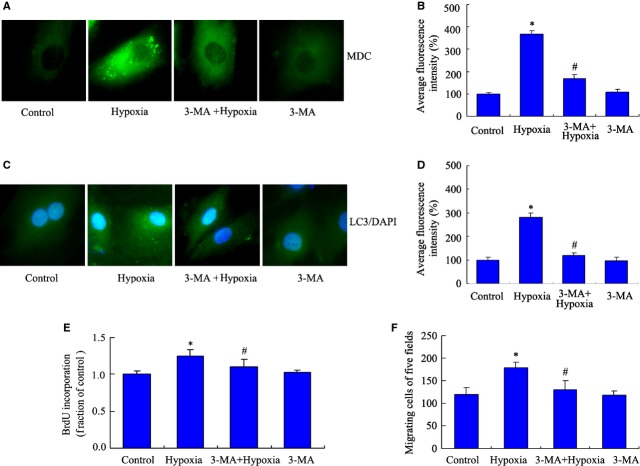
3-MA inhibits autophagy and decreases the proliferation of pulmonary arterial smooth muscle cells (PASMCs) induced by hypoxia. PASMCs were pre-incubated with 3-MA (5 mM) for 30 min. after 24 hrs, cells were exposed to hypoxia and normoxia chamber for 24 hrs. (A) The formations of autophagic vacuoles were detected by punctated monodansylcadaverine (MDC) immunofluorescence staining. Microphotographs are shown as representative results from three independent experiments. Images are at 1000×. (B) The corresponding linear diagram of MDC staining results. (C) PASMCs were processed for LC3 immunofluorescence staining. (D) The corresponding linear diagram of LC3 staining. (E) Cell proliferation was measured by 5-bromo-2′-deoxyuridine (BrdU) assay. *n* = 5, mean ± SD. *P < 0.05 *versus* control group, ^#^*P* < 0.05 *versus* hypoxia group. (F) Migration of PASMCs exposed to 3-MA under hypoxia was detected by transwell assay. *n* = 5, mean ± SD. *P < 0.05 *versus* control group, ^#^*P* < 0.05 *versus* hypoxia group.

To identify the role of autophagy in PASMCs induced by hypoxia, an autophagy-specific inhibitor, 3-MA, was added into our hypoxia cell model *in vitro*. This inhibitor has no significant toxic effect in certain cells including SMCs [33–35]. Autophagic vacuoles were detected by MDC immunofluorescence staining. Compared with the hypoxia group at 24 hrs, the group exposed to 5 mM 3-MA presented decreased accumulation of autophagic vacuoles, which indicates that 3-MA inhibited the autophagy induced by hypoxia (Fig. 3A and B). Subsequently, we analysed the formation of LC3 puncta using LC3 immunofluorescence staining, and found consistent results with MDC immunofluorescence staining (Fig. 3C and D). In addition, cell proliferation and migration were also measured as described above. Our results indicated that the addition of 3-MA decreased PASMCs proliferation and migration at 24 hrs under hypoxia (Fig. 3E and F), which suggest that autophagy may be essential for PASMC proliferation under hypoxia.

### Apelin decreases proliferation and migration *via* inhibiting autophagy in PASMCs under hypoxia

**Figure 4:**
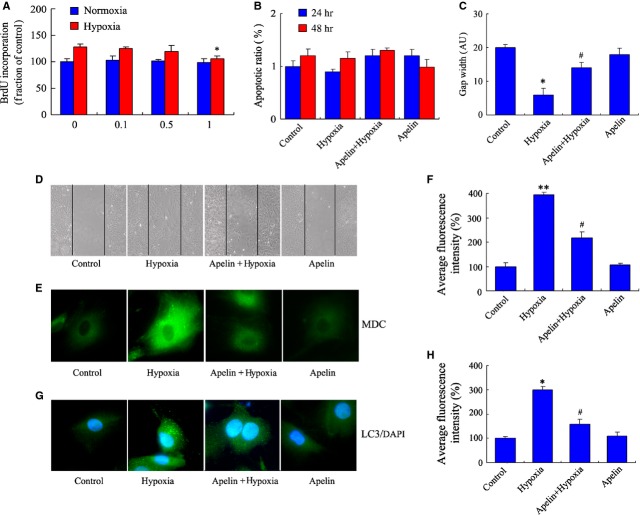
Apelin decreases the proliferation and migration *via* inhibiting autophagy in pulmonary arterial smooth muscle cells (PASMCs) under hypoxia. (A) PASMCs were pre-incubated with different concentrations (0.1, 0.5 and 1 μM) apelin for 30 min., and then exposed to hypoxia chamber and normoxia chamber for 24 hrs; cell proliferation was measured by 5-bromo-2′-deoxyuridine (BrdU) assay. *n* = 5, mean ± SD. *P < 0.05 *versus* control group. (B) The apoptosis rate of PASMCs in hypoxia condition, which was pre-incubated with 1 μM apelin for 30 min. and then placed in 1% oxygen for 24 or 48 hrs. (C) Apelin inhibited cell migration of PASMCs in hypoxia condition. PASMCs were pre-incubated with apelin and then placed in 1% oxygen for 24 hrs; scratches were made with a pipette tip. The widths of scratched gaps were measured. *P < 0.05 *versus* control group, #*P* < 0.05 *versus* hypoxia group. *n* = 5. (D) Cell migration and representative pictures of PASMCs were taken at different conditions. (E) Effect of apelin on autophagy in PASMCs under hypoxia. PASMCs were labelled with monodansylcadaverine (MDC) and observed with a fluorescent microscope. Images are at 1000×. Microphotographs were shown as representative results from three independent experiments. (F) The corresponding linear diagram of MDC staining results. **P < 0.01 *versus* control group, ^#^*P* < 0.05 *versus* hypoxia group. (G) Representative images of PASMCs were stained with DAPI (blue), and antibodies against LC3 (green), punctuated LC3 dots were considered as positive results. Images are at 1000×. (H) The corresponding linear diagram of LC3 staining. *P < 0.05 *versus* control group, ^#^*P* < 0.05 *versus* hypoxia group.

We next examined the effect of exogenous apelin in the proliferation of PASMCs. Cells were treated with different concentrations (0.1, 0.5 and 1 μM) of apelin and then placed for 24 hrs in the hypoxia chamber and normoxia chamber. Cell migration was also initially detected with a transwell assay. Our results demonstrated that different concentrations of apelin have no significant effect on the proliferation of PASMCs under normoxia conditions (*P *>* *0.05, Fig. 4A). In addition, 1 μM apelin decreased PASMC proliferation under hypoxia conditions at 24 hrs as compared with the control group (*P* < 0.05, Fig. 4A). Moreover, the apoptosis of PASMCs under hypoxia was also determined by FACScan; there was no obvious apoptosis both in 24 and 48 hrs hypoxia groups whether treated with apelin or not (*P* > 0.05, Fig. 4B). The effect of apelin on the migration of PASMCs was additionally investigated using a wound healing assay. Pictures of the scratched wounds were taken at 0 and 24 hrs. It was observed that the wound width of the scratched gaps decreased markedly, suggesting that apelin administration significantly inhibited PASMC migration under hypoxia as compared with the hypoxia control group (*P* < 0.05, Fig. 4C and D).

To investigate whether the role of apelin is related to the regulation of autophagy in PASMC proliferation under hypoxia, PASMCs were treated with apelin for 24 hrs under hypoxia or normoxia conditions. Our data indicated that apelin treatment decreased the accumulation of MDC-positive dots in PASMCs under hypoxia (Fig. 4E and F). We further observed the autophagic marker LC3 expression by immunofluorescence staining, which is consistent with the results of MDC staining. The formation of LC3 puncta decreased significantly, indicating that apelin inhibited autophagy of PASMCs under hypoxia (Fig. 4G and H).

### Activation of PI3K/Akt/mTOR pathways is involved in the regulation of autophagy by apelin treatment in PASMCs under hypoxia

**Figure 5:**
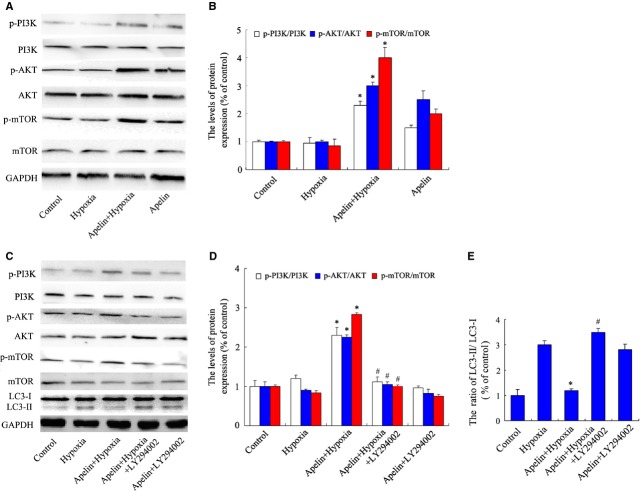
The effect of apelin on autophagy in pulmonary arterial smooth muscle cells (PASMCs) induced by hypoxia is related to the regulation of PI3K/Akt/mTOR pathways. (A) apelin increases the phosphorylation of PI3K/Akt/mTOR signals. The protein expressions were measured by western blot analysis. (B) Densitometry was applied to quantify the protein density. Standard error represents three independent experiments. *P < 0.05 *versus* hypoxia group. (C) Expression of phosphorylated-PI3K/Akt/mTOR and LC3 protein in PASMCs under hypoxia with apelin and Akt inhibitor LY294002. (D) Densitometry was applied to quantify phospho-PI3K/AKT/mTOR protein density. *P < 0.05 *versus* hypoxia group, ^#^*P* < 0.05 *versus* apelin-treated hypoxia group. (E) The ratio of normalized LC3-II to LC3-I; the data were presented as a mean ± SD from three independent experiments. *P < 0.05 *versus* hypoxia group, ^#^*P* < 0.05 *versus* apelin-treated hypoxia group.

Our next goal was to demonstrate whether the decrease in autophagy induced by apelin was dependent on the regulation of PI3K/Akt/mTOR pathways. After apelin treatment for 24 hrs under hypoxia, the levels of phosphorylated PI3K, Akt and phosphorylated mTOR were up-regulated under hypoxia (Fig. 5A and B). To further confirm whether the role of apelin is PI3K/Akt-signal dependent, the classic pathway inhibitor LY294002 was added together with apelin in PASMCs under hypoxia. As shown in Figure 5C and D, LY294002 blocked the activation of Akt and downstream mTOR signals, compared with the apelin-treated hypoxia group. Moreover, the effect of apelin on autophagic protein was determined by western blot analysis. The expression of LC3-II was inhibited by apelin treatment at 24 hrs induced by hypoxia, compared with the untreated hypoxia group. The addition of LY294002 markedly increased the expression of LC3-II compared with the apelin-treated hypoxia group, and partially abolished the inhibition of autophagy associated with apelin treatment (Fig. 5C and E). These data revealed that a bypassing mechanism of PI3K/Akt signalling targets autophagy inhibition dependent on mTOR suppression, which may be involved in facilitating the effects of apelin treatment on the proliferation of PASMCs.

### Apelin activates Akt/mTOR signalling, inhibits autophagy and is APJ-receptor dependent in PASMCs under hypoxia

**Figure 6:**
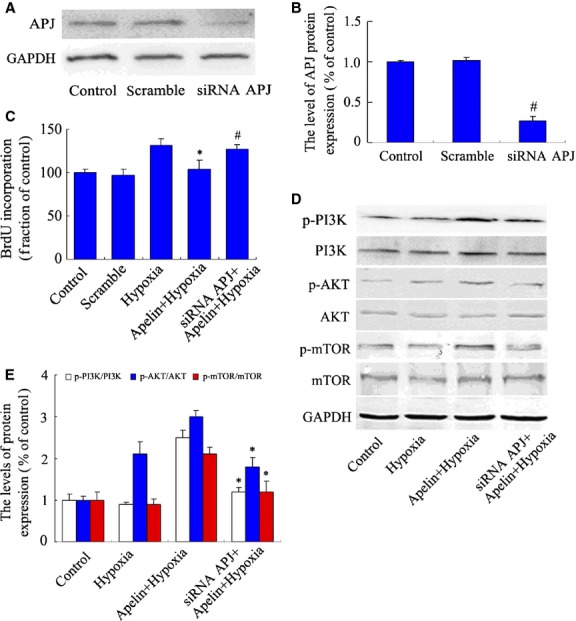
The effect of siRNA-APJ on the proliferation and activation of PI3K/Akt/mTOR signals in pulmonary arterial smooth muscle cells (PASMCs) under hypoxia. (A) Western blot analysis of APJ receptor protein expression in PASMCs transfected with siRNA-APJ and scramble vectors as described above for 24 hrs. (B) Densitometry was applied to quantify the protein density. Data were presented as a mean ± SD from three independent experiments. ^#^*P* < 0.01 *versus* scramble group. (C) PASMCs treated with siRNA-APJ and scramble siRNA vectors for 24 hrs, cell proliferation was measured by 5-bromo-2′-deoxyuridine (BrdU) assay. *P < 0.05 *versus* hypoxia group. ^#^*P* < 0.05 *versus* apelin-treated hypoxia group. *n* = 5. (D) Phosphorylation of PI3K/Akt/mTOR protein in PASMCs treated with siRNA-APJ and apelin in hypoxia condition. (E) Densitometry was applied to quantify the protein density; data were presented as a mean ± SD from three independent experiments. *P < 0.05 *versus* apelin-treated hypoxia group.

**Figure 7:**
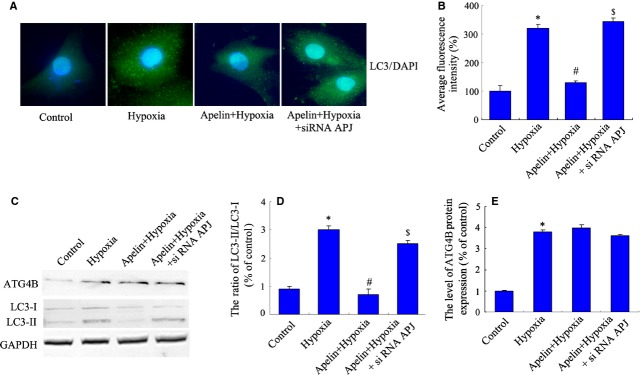
Transfection of siRNA-APJ blocks the inhibitory effect of apelin on autophagy in pulmonary arterial smooth muscle cells (PASMCs) under hypoxia. PASMCs treated with apelin and transfected with siRNA-APJ in hypoxia conditions. (A) Representative images of PASMCs were stained with DAPI (blue) and antibodies against LC3 (green). Images are at 1000×. Microphotographs were shown as representative results from three independent experiments. (B) The corresponding linear diagram of LC3 staining. (C) The protein levels of ATG4B and LC3 were detected with immunoblotting. (D) The ratio of normalized LC3-II to LC3-I. Data were presented as a mean ± SD from three independent experiments. *P < 0.05 *versus* control group, ^#^*P* < 0.05 *versus* hypoxia group, ^$^*P* < 0.05 *versus* apelin-treated hypoxia group. (E) The ratio of normalized ATG4B protein. Data were presented as a mean ± SD from three independent experiments. *P < 0.05 *versus* control group.

To further confirm the role of the apelin-APJ system in the autophagy and cell proliferation of PASMCs under hypoxia, PASMCs were transfected with siRNA-APJ and scrambled siRNA vectors as described above. The transfection of scrambled siRNA had no obvious effect on the expression of APJ. The siRNA-APJ vector inhibited the expression of APJ protein to 27% in PASMCs, compared with the scrambled siRNA group (Fig. 6A and B). In the BrdU incorporation assay, cell proliferation does not obviously change in scramble group, compared with the normoxia control group. Exogenous apelin did not suppress cell proliferation of APJ-deficient cells under hypoxia, compared with the apelin-treated hypoxia group (Fig. 6C). The suppression of APJ abolished the apelin-induced activation of PI3K/Akt/mTOR, and the phosphorylation of PI3K/Akt/mTOR decreased significantly following siRNA transfection (Fig. 6D and E). Furthermore, in LC-3 immunofluoresence staining (Fig. 7A and B) and protein level analysis (Fig. 7C and D), siRNA-APJ also abolished the inhibition effect of autophagy by exogenous apelin in PASMCs cultured in hypoxic conditions. Both apelin treatment and siRNA-APJ have no effect on the protein expression of ATG4B (cleaving the LC3 C-terminal domain to generate LC3-I, Fig. 7C and E), suggested that the effect of apelin may related to the formation of LC-3II, but not upstream cysteine protease. All of these results indicate that the role of apelin in the autophagy regulation is APJ-receptor dependent in PASMCs under hypoxia.

## Discussion

Hypoxic pulmonary hypertension is characterized by a progressive increase in pulmonary vascular resistance, which includes clinical symptoms such as dyspnoea, cyanosis and acute, right-sided heart failure [36]. One trigger of HPH is hypoxia, which acutely causes a significant increase in pulmonary blood pressure by vasoconstriction, but chronically results in the structural remodeling of the pulmonary vasculature [37,38]. A number of vasoactive factors have been described as playing important roles in the progression of HPH in both experimental and clinical settings, yet little is known about the cellular and molecular causes of HPH [39,40]. In general, pulmonary arterial changes have been considered to be caused by the proliferation of cells with the characteristics of SMCs. Therefore, one effective treatment for HPH may rely on the development of novel strategies for inhibiting SMCs proliferation [41,42].

In previous studies, the activation of autophagy has been demonstrated to be involved in the process of HPH, acute pulmonary disease *in vivo* and cell models treated with hypoxic conditions *in vitro* [43,44]. Increases of autophagy levels were detected in clinical samples of human lung tissue from patients with chronic obstructive pulmonary disease (COPD) and in mouse lung tissue subjected to chronic cigarette smoke exposure (CSE), in addition to pulmonary cells exposed to cigarette smoke extract [45]. Cigarette smoke exposure increases the processing of LC3-I to LC3-II in cigarette smoke–induced COPD. Inhibition of autophagy by LC3B knockdown protects arterial epithelial cells from CSE-induced apoptosis. In Egr-1 (whose expression changes significantly in COPD)–deficient mice, resist cigarette smoke induced autophagy, apoptosis and emphysema, suggesting that autophagy provides a protective effect in CSE-induced COPD [46]. In the latest study, chloroquine inhibits autophagy and blocks lysosomal degradation of the bone morphogenetic protein type II receptor, inhibiting proliferation and increased apoptosis of PASMCs in established HPH models both *in vivo* and *in vitro* [47]. In our study, we demonstrated that activation of autophagy is involved in the PASMC proliferation and migration induced by hypoxia, and inhibition of autophagy by the specific inhibitor resulted in a decrease in cell proliferation and cell cycle arrest, suggesting that the increase in autophagy stimulated PASMCs proliferation in the hypoxia condition, which may function as an important mediator of disease progression and the development of arterial remodeling in HPH.

It is worth to mention that autophagy is either an adaptive necessary process or potentially deleterious. In different cells, different conditions or stress, autophagy may play converse functions in the process of cell death or pathophysiology of diseases, to figure out the threshold is benefit of the outcome for further exploration. Hypoxic pulmonary hypertension is a special disease with pulmonary remodeling including proliferation of arterial SMCs (PASMCs) and injury of endothelium cells. To block the proliferation and migration but not induce cell death of PASMCs is one of the key strategies in the therapy of HPH [48,49]. In our study, we have detected the effect of hypoxia in the apoptosis of PASMCs, and did not find significant apoptosis even after 48 hrs of hypoxia exposure. This suggested that in the early stage of our cell model under hypoxia, the role of autophagy is an adaptive process, which increases the proliferation and migration of PASMCs, and the beneficial effect of apelin may play an inhibitory function on autophagy *via* activation of downstream signals. Nevertheless, as a dual physiological process, the role of autophagy also related to cell death, but probably activates the cell death of endothelium cells in HPH, which still need to further investigations. Collectively, the strategy with apelin on regulation of autophagy in PASMCs under hypoxia should target on how to inhibit autophagy mandatory to a natural restoration but not tuned.

One of the first proven physiological effects of apelin is the ability to temporarily lower blood pressure after injection in rats. This effect was further confirmed in human volunteers and heart failure patients in several other studies [22,50]. In addition, two studies have shown that serum apelin levels in patients with HPH are lower than in controls. Another finding was that apelin inhibits platelet-derived growth factor B–mediated proliferation and triggers apoptosis in PASMCs [22,51]. These studies support a definite role of apelin in pulmonary hypertension, although the underlying mechanism still requires further investigation. Recent studies have explored a potential role for augmentation of apelin signalling in ameliorating rodent models of pulmonary hypertension [52,53]. Mice lacking the apelin gene develop worsening HPH in response to hypoxia, suggesting that the level of apelin may be involved in the process of HPH. Injections of exogenous apelin of wild HPH mice resulted in the reversal of right ventricular systolic pressure, hypertrophy and muscularization of alveolar wall pulmonary arteries [51]. In our study, apelin inhibited the increase in cell proliferation and blocked the cell cycle progression of PASMC responses to hypoxia, and decreased the level of autophagy under hypoxia, suggesting that the role of apelin in the regulation of PASMCs may be related to the inhibition of autophagy in the HPH cell model *in vitro*. In a recent study, treatment with the autophagy inhibitor chloroquine prevented proliferation and increased apoptosis of cultured rat PASMCs *via* inhibiting autophagy pathways [47], which is consistent with our results. Moreover, it should be considered that the mechanisms of autophagy inhibitors such as chloroquine or 3-MA are different from apelin in regulation of autophagy. To block the lysosomal degradation or formation of autophagic double membrane structures may lead to diverse consequences under specific stress. Collectively, our study and the other studies with these classic inhibitors in PASMCs *in vitro* or *in vivo* illustrated a clue that as an endogenous protein, which is highly expressed in the lungs, apelin exerts beneficial effects that may be involved in the inhibition of autophagy in experimental HPH.

Apelin has been shown to promote the activation of the phospho-Akt pathway, which plays important roles in physiological functions [48,54], suppress human osteoblasts apoptosis *via* the APJ/PI3K/Akt signalling pathway [55]. Apelin attenuates the differentiation of cultured calcifying VSMCs, which are considered a model for the study of vascular calcification, and additions of the PI3K inhibitor LY294002 and APJ siRNA reversed the effects of apelin [56]. On the other hand, it is well known that as upstream pathways, PI3K/Akt/mTOR signals are essential for the regulation of autophagy [57–59], but the role of autophagy and PI3K/Akt/mTOR in PASMCs of HPH experimental models has not been discussed. In this study, we firstly demonstrated that the activation of PI3K/Akt/mTOR is essential for the effect of apelin on autophagy under hypoxia. Furthermore, suppression of APJ with siRNA blocked the activations of Akt and downstream signalling of mTOR by apelin, reversing the proliferation and autophagy of PASMCs. These data *in vitro* indicated the regulation of autophagy and downstream signals by apelin maybe related to the effect of apelin, which is beneficial to inhibition of vascular remodeling in HPH. To the best of our knowledge, the limitation of this study also suggests that it is definitely necessary to explore further investigation and research *in vivo*, by apelin-deficient mice or apelin treatment to confirm the effect of apelin *in vivo*, and investigates the role of apelin in the remodeling of pulmonary arterial vascular and the relations to HPH, which may support apelin as therapeutic targets or strategies for HPH in further clinical trials or explorations.

In conclusion, our study demonstrates that hypoxia induced the proliferation and migration of PASMCs through the activation of autophagy. Inhibition of autophagy by the autophagic specific inhibitor decreases the proliferation of PASMCs. In addition, exogenous apelin inhibited autophagy and decreased cell proliferation through the activation of the PI3K/Akt/mTOR signalling pathway, which is APJ-receptor dependent. This study provides novel evidence that exogenous apelin treatment may provide potential strategy by inhibiting autophagy in the proliferation of PASMCs, which is essential for the arterial remodeling process of HPH.
